# Supplementary material for: Microfluidic droplet application for bacterial surveillance in fresh-cut produce wash waters
Source: PLoS One. 2020 Jun 9;15(6):e0233239. doi: 10.1371/journal.pone.0233239 (PMC7282644; doi:10.1371/journal.pone.0233239)
Supplement: S1 Table — (DOCX) [file pone.0233239.s001.docx]

**SI Table 1**: Relative Fluorescence of Bacteria to Background In-Droplet Grown in Rappaport-Vassiliadis Broth Over 5-Hour Incubation at 37°C

| *Bacteria* | *0 (hr)* | *1* | *2* | *3* | *4* | *5* |
| --- | --- | --- | --- | --- | --- | --- |
| *S.* Typhimurium | 3.20*^a^* (0.78)*^b^*,  2.51-3.88*^c^* | 3.05 (0.65),  2.48-3.62 | 3.52 (0.31),  3.25-3.80 | 2.61 (0.76),  1.95-3.28 | 2.36 (0.24),  2.15-2.58 | 2.38 (0.47),  1.97-2.80 |
| *E. aerogens* | 1.22 (0.06),  1.16-1.28 | 1.18 (0.07),  1.12-1.24 | 1.30 (0.14),  1.18-1.42 | 1.12 (0.04),  1.08-1.16 | 1.12 (0.04),  1.09-1.16 | 1.24 (0.15),  1.11-1.37 |
| *E. coli 700609* | 1.11 (0.03),  1.08-1.13 | 1.13 (0.04),  1.10-1.16 | 1.13 (0.09),  1.05-1.08 | 1.13 (0.05),  1.08-1.17 | 1.13 (0.04),  1.09-1.17 | 1.29 (0.05),  1.25-1.34 |
| *E. coli 13706* | 1.11 (0.03), 1.08-1.13 | 1.13 (0.07), 1.06-1.19 | 1.13 (0.05), 1.09-1.18 | 1.13 (0.05), 1.08-1.17 | 1.13 (0.07), 1.07-1.19 | 1.29 (0.13), 1.18-1.41 |
| *E. coli 700891* | NFD*^d^* | NFD | NFD | 1.05 (0.02), 1.04-1.07 | 1.05 (0.02), 1.03-1.07 | 1.10 (0.04), 1.06-1.13 |
| *C. freundii* | NFD | NFD | NFD | 1.05 (0.02), 1.04-1.06 | 1.04 (1.01), 1.03-1.05 | 1.09 (0.05), 1.04-1.13 |

*a: Mean (n=5); b: Standard Deviation; c: 95% Confidence Interval; d: No Fluorescence Detection (NFD)*
