# Supplementary material for: Microfluidic droplet application for bacterial surveillance in fresh-cut produce wash waters
Source: PLoS One. 2020 Jun 9;15(6):e0233239. doi: 10.1371/journal.pone.0233239 (PMC7282644; doi:10.1371/journal.pone.0233239)
Supplement: S2 Fig — Adjusted fluorescence measurements of 0.1, 1, and 10 μg/ml FITC in each media with four replicates. The grey trend line represents the PBS control to better display fluorescent contribution of media, and the fluorescent masking effect of RV and/or pH-sensitivity of FITC at low pH. (DOCX) [file pone.0233239.s003.docx]

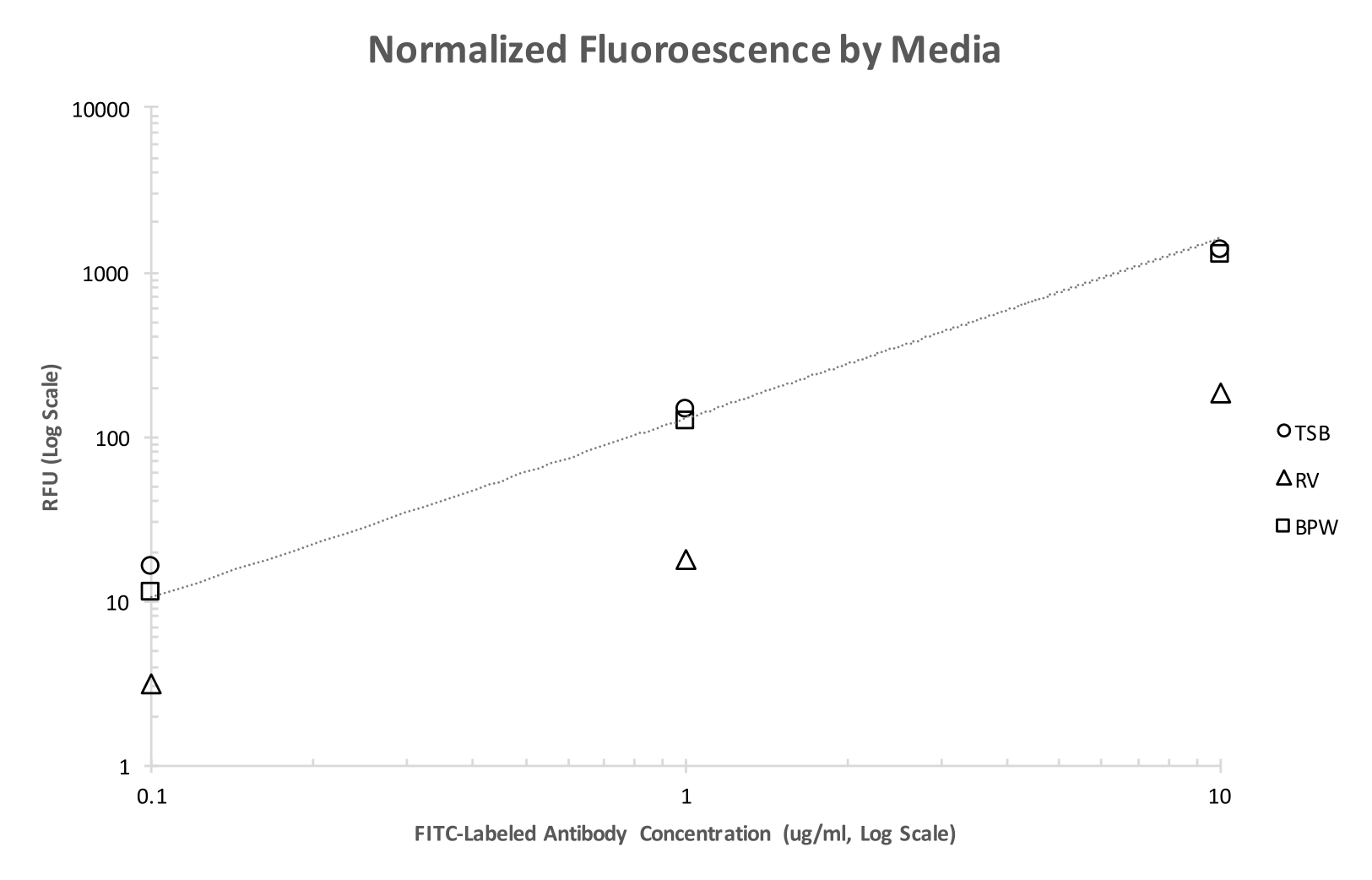


**SI Figure 2**: TSB = Tryptic Soy Broth; BPW = Buffered Peptone Water; RV = Rappaport-Vassiliadis Broth; PBS = Phosphate Buffered Saline. Adjusted fluorescence measurements of 0.1, 1, and 10 µg/ml FITC in each media with four replicates. The grey trend line represents the PBS control to better display fluorescent contribution of media, and the fluorescent masking effect of RV and/or pH-sensitivity of FITC at low pH.
