# Supplementary material for: Microfluidic droplet application for bacterial surveillance in fresh-cut produce wash waters
Source: PLoS One. 2020 Jun 9;15(6):e0233239. doi: 10.1371/journal.pone.0233239 (PMC7282644; doi:10.1371/journal.pone.0233239)
Supplement: S3 Fig — A total of five fluorescence values (n = 5) were analyzed for each media and the PBS control. Medias include Rappaport-Vassiliadis broth (RV), buffered peptone water (BPW), and tryptic soy broth (TSB). Direct fluorescence measurements correspond with the left y-axis, and relative fluorescence measurements correspond with the right y-axis. Error bars represent the 95% confidence interval. (DOCX) [file pone.0233239.s004.docx]

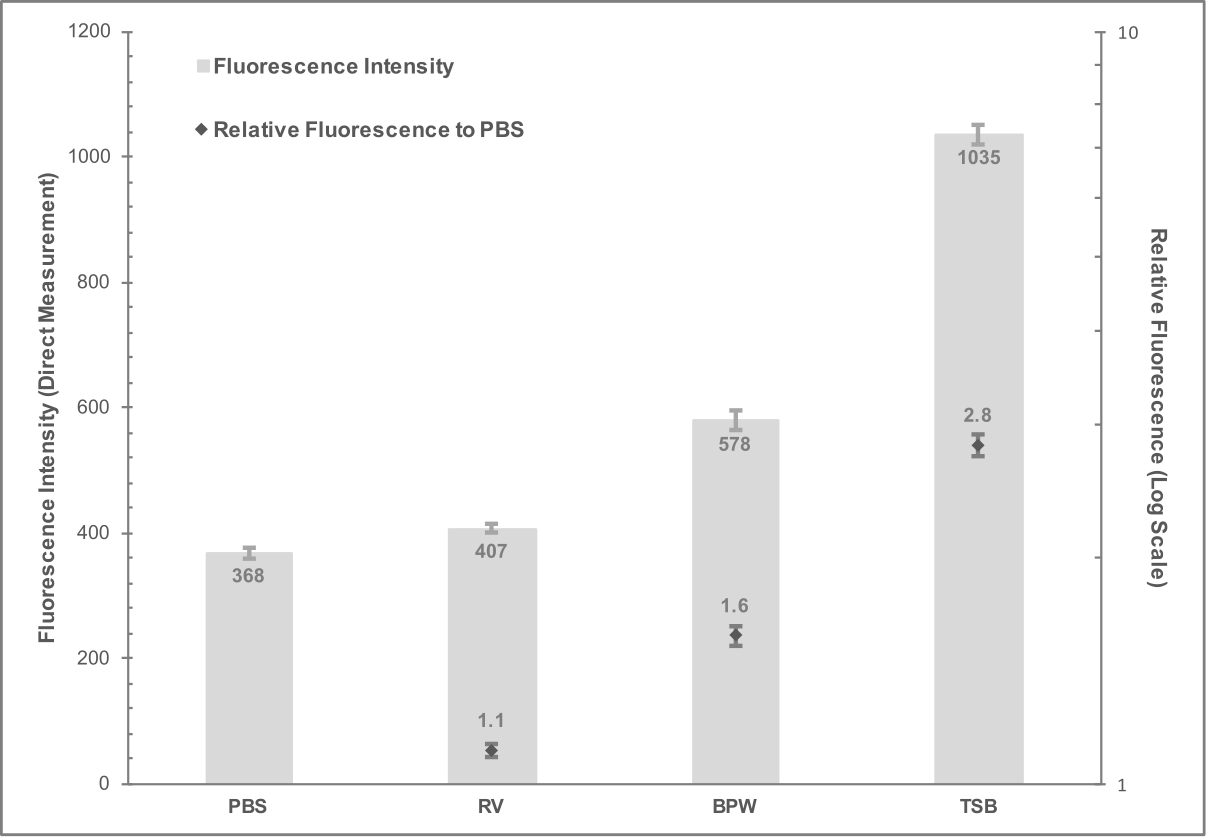


**SI Figure 3**: Direct fluorescence intensity measurements and relative fluorescence to phosphate buffered saline (PBS) of each media-FITC-Ab data image in Figure 6. A total of five fluorescence values (n=5) were analyzed for each media and the PBS control. Medias include Rappaport-Vassiliadis broth (RV), buffered peptone water (BPW), and tryptic soy broth (TSB). Direct fluorescence measurements correspond with the left y-axis, and relative fluorescence measurements correspond with the right y-axis. Error bars represent the 95% confidence interval.
