# Supplementary material for: Microfluidic droplet application for bacterial surveillance in fresh-cut produce wash waters
Source: PLoS One. 2020 Jun 9;15(6):e0233239. doi: 10.1371/journal.pone.0233239 (PMC7282644; doi:10.1371/journal.pone.0233239)
Supplement: S4 Fig — Bacterial strains (a: E. coli 700609; b: E. coli 13706; c: E. coli 700891) with 0.83 μg/ml FITC-Ab in phosphate buffered saline.) each droplet is approximately 50–70 μm in diameter (scale on image is 50 μm). Bright field images are presented complimentary to FITC images and visually show bacterial concentration at an ideal focal plane (5 μm interval stacking and merging of images into two dimensional images is not optimal for bright field images). FITC images are merged 5 μm interval focal plane images and represent a two-dimensional image of the entire droplet. (DOCX) [file pone.0233239.s005.docx]

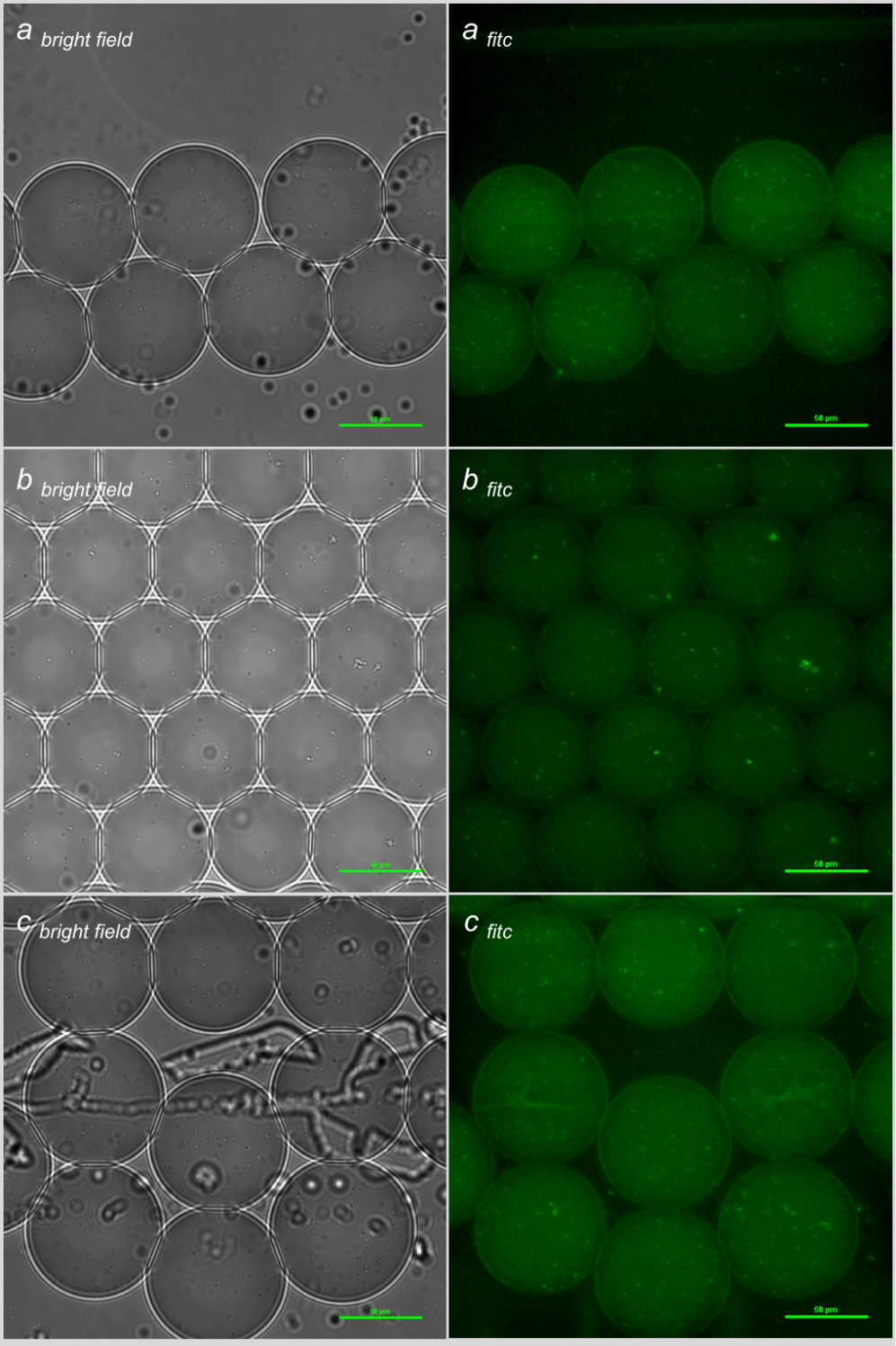


**SI Figure 4**: Bacterial strains (a: *E. coli* 700609; b: *E. coli* 13706; c: *E. coli* 700891) with 0.83 µg/ml FITC-Ab in phosphate buffered saline.) Each droplet is approximately 50-70 µm in diameter (scale on image is 50 µm). Bright field images are presented complimentary to FITC images and visually show bacterial concentration at an ideal focal plane (5 µm interval stacking and merging of images into two dimensional images is not optimal for bright field images). FITC images are merged 5 µm interval focal plane images and represent a two-dimensional image of the entire droplet.
