# Supplementary material for: Microfluidic droplet application for bacterial surveillance in fresh-cut produce wash waters
Source: PLoS One. 2020 Jun 9;15(6):e0233239. doi: 10.1371/journal.pone.0233239 (PMC7282644; doi:10.1371/journal.pone.0233239)
Supplement: S5 Fig — The trend line represents a decrease in relative fluorescence of S. Typhimurium over the time course. A detection threshold region is identified on the figure. No measurable relative fluorescence was identified in time points 0 through 2 for both C. freundii and E. coli 700891. Five replicates were measured for each bacterial species/strain. (DOCX) [file pone.0233239.s006.docx]

**
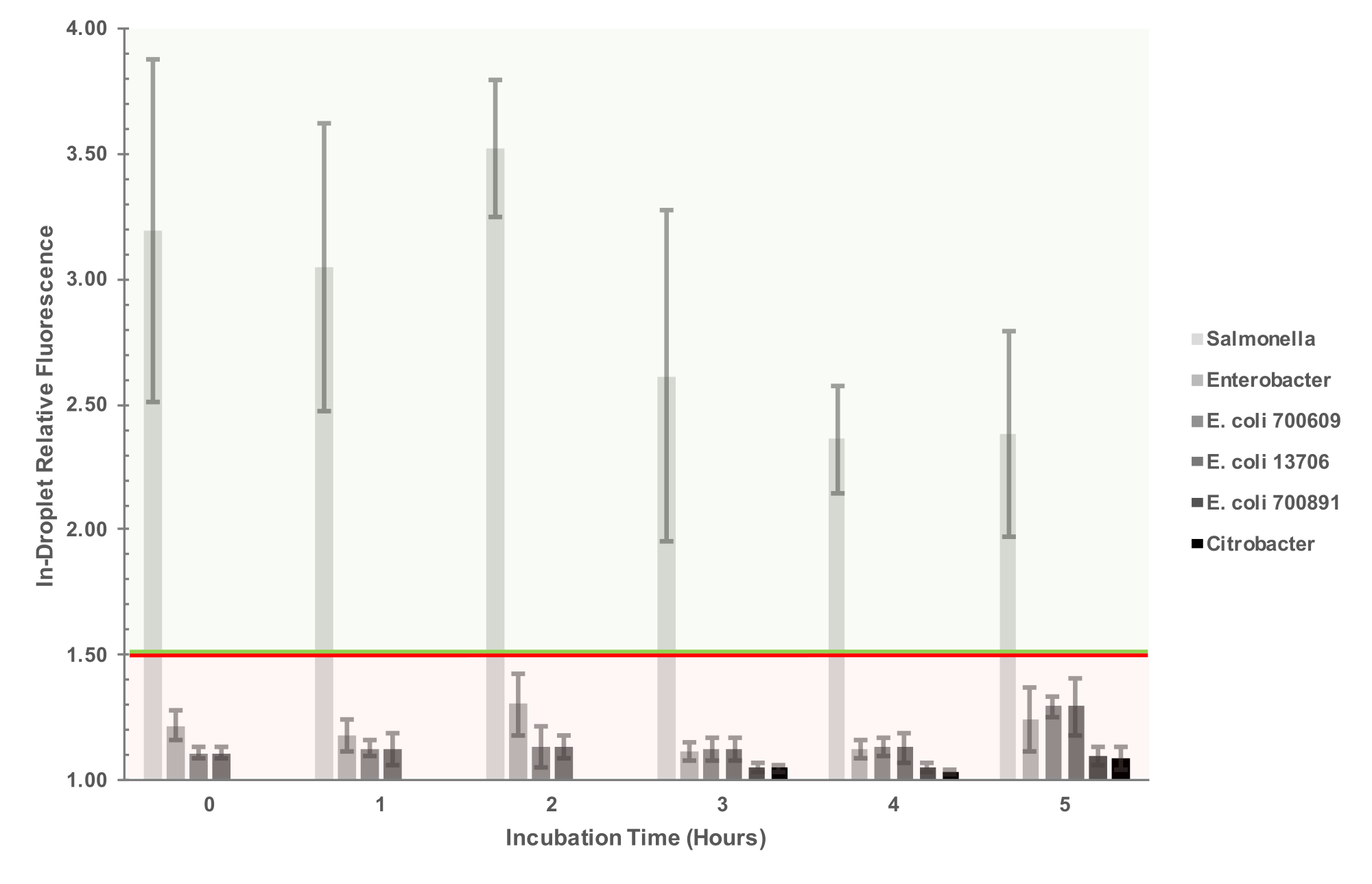
**

**SI Figure 5**: Relative fluorescence of bacterial species (*S.* Typhimurium, *E. aerogens*, and *C. freundii*) and strains (*E. coli* 700609, 13706, and 700891) incubated in Rappaport-Vassiliadis broth for five hours at 37°C with a FITC-Ab concentration of 10 µg/ml in-droplet. The trend line represents a decrease in relative fluorescence of *S.* Typhimurium over the time course. A detection threshold region is identified on the figure. No measurable relative fluorescence was identified in time points 0 through 2 for both *C. freundii* and *E. coli* 700891. Five replicates were measured for each bacterial species/strain.
