# Supplementary material for: Microfluidic droplet application for bacterial surveillance in fresh-cut produce wash waters
Source: PLoS One. 2020 Jun 9;15(6):e0233239. doi: 10.1371/journal.pone.0233239 (PMC7282644; doi:10.1371/journal.pone.0233239)
Supplement: S11 Fig — (DOCX) [file pone.0233239.s012.docx]

**
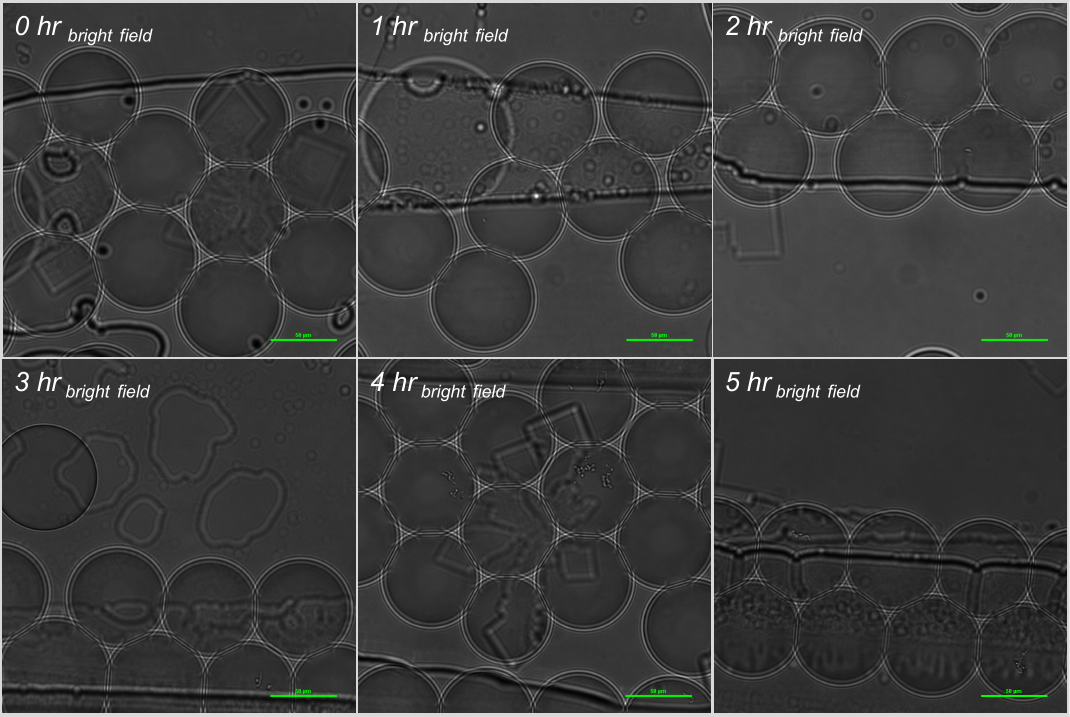
**

**SI Figure 11**: Bright field images of *E. coli* 13706 incubation in-droplet with sterile deionized water, and 10 µg/ml FITC-Ab in 1x RV broth at 37°C.
